# Role-Play Experience Facilitates Reading the Mind of Individuals with Different Perception

**DOI:** 10.1371/journal.pone.0074899

**Published:** 2013-09-04

**Authors:** Fumikazu Furumi, Masuo Koyasu

**Affiliations:** 1 Graduate School of Education, Kyoto University, Kyoto, Japan; 2 Japan Society for the Promotion of Science, Tokyo, Japan; University of Milan, Italy

## Abstract

The present study examined effects of role-play experience on reading the mind of people with different perception. It is normally difficult but very important in daily life to understand people with different characteristics, including those with restricted color vision. We explored the mechanisms of reading the mind of people with different perception. Forty university students were introduced to a communication task in which the use of mindreading was essential. During each trial, participants viewed a shelf, presented on a laptop computer, which contained several familiar objects, and they were instructed to touch an object on the shelf following an instruction issued by a partner who stood at the opposite side of the shelf. There were two partners: one was a monkey with normal color vision and the other was a dog with restricted color vision. The monkey could see all the objects in the same colors as the participants, whereas the dog saw some objects in different colors (e.g., he saw as yellow objects that the participants saw as red). Participants were required to respond according to the partner's instruction. In the restricted color vision condition, the dog saw the colors of objects differently; thus, participants had to work out his intentions (i.e., mind read), according to his different perspective. In the normal color vision condition, all objects were in the same colors as those seen by the monkey. Before the test phase, the role-play group had a role-play experience in which participants assumed the role of people with restricted color vision. No-role-play participants made significantly more errors in the restricted color vision condition than in the normal color vision condition, whereas among role-play participants, there was no difference between conditions. These results suggest that role-play experience facilitates reading the mind of people with perceptual experiences different from our own.

## Introduction

Many studies about mindreading have been conducted on preschoolers and primary school children (e.g., [1]–[2]). On the other hand, some studies about adults' mindreading have been gradually conducted recently (e.g., [3]–[7]). For example, Dumontheil, Apperly, and Blakemore [4] modified Kyesar's Director Task [3] to investigate a development of mindreading after adolescence. In the Director Task, participants viewed a shelf with 4×4 slots, presented on a laptop computer, which contained several familiar objects, and were instructed to move one of the objects on the shelf following an instruction issued by a “director” who stood at the opposite side of the shelf. Although the director could not always see all of the objects, the rules stipulated that all instructions involved objects that were visible to the director. Under the experimental condition, the most suitable target was not visible to the director, presumably leading participants not to move the most appropriate object. For instance, when there were three different cups on the shelf (the smallest one was in the occluded slot), and the director asked a participant to move the small cup, the correct response was to move the middle size cup. This was because the smallest one was invisible to the director. Participants had to consider the mind of the director. Under the control condition, the most suitable object was visible to the director. For example, when the director asked a participant to take the small cup when there were two different cups on the shelf (both in opened slots), the correct response was to move the smaller one. This is because both were visible to him. Using this task, they found that mindreading continues to develop in late adolescence. Dumontheil, Küster, et al. [5] suggested that such a Director Task involves level 1 perspective taking [8].

Apperly [9] suggested that experience is needed for mindreading development. Apperly reviewed papers which argued the relationships between mindreading ability and social experience. Furumi and Koyasu [10] investigated the effect of role-play as a social experience. In this study, exploring mindreading in adults, participants had an experience of playing another's role. Furumi and Koyasu found that experience with role-play facilitated mindreading. They modified the Director Task and allocated participants two groups: role-play and no-role-play. The role-play group played the director's role before the task, whereas, the no-role-play group only heard the instructions. It was found that the role-play group made fewer errors than the no-role-play group and could respond more quickly than the no-role-play group.

Many studies about adults' mindreading have used difficult tasks like Keysar's Director Task, in these difficult tasks, the situation creates a scene requiring complex mindreading, where the participant's own experience is helpful and acts as a cue. Komeda, Kawasaki, Tsunemi, and Kusumi [11] suggested that people can easily understand others' minds when they try to read the minds of those who are similar to themselves. In their study, participants read the story and rated the protagonist's emotional states. The results showed that extraverted participants rated correctly when the protagonist was similar to themselves. Komeda, Kosaka, Saito, Inohara, Munesue, et al. [12] found that autistic people can read and memorize stories with an autistic protagonist more easily than that with a typically developing protagonist. On the other hand, typically developing people can read and memorize the story with a typically developing protagonist more easily than that with autistic protagonist. This suggests that people have difficulty in understanding others with different characteristics.

Executive function skills are also important for taking perspectives of others especially if they have different characteristics. This is because we have to inhibit our egocentric perspectives when we try to work out the different mental states of others [9]. Lin, Keysar, and Epley [13] suggested that we are reflexively mindblind because lots of cognitive resources are required for mindreading. In their study, performance on the Director Task was worse in the high cognitive load condition than in the low cognitive load condition. Moreover, individual differences of executive function skill (working memory capacity) affect performance on the Director Task.

In this study, we developed a modified Director Task, in which a communication partner has a different perception in order to investigate the effect of role-play on mindreading among people who have a different cognitive experience (the Director and the participant). Participants cannot use their own experience as a cue because they do not have the same experience as that of a communication partner (the Director) who has a different cognitive experience. Because of this, this novel type of Director Task involves level 2 perspective taking [8].

Color vision defect is a characteristic that most people do not experience. Since people with color vision defects perceive a differently colored world than people with normal color vision, their cognitions are supported by different color perceptions than people with normal color vision. Congenital red green color vision defect is caused by sex-linked recessive inheritance. Because of this, there are more male congenital red green color vision defect people than females. Birch [14] shows that 4–8% of males have congenital red green color defect. In addition, it has been claimed that there are differences between ethnic groups in the prevalence of red green color defect. Although people with color vision defect have few difficulties in daily life, there is different color representation between color vision defect people and normal color vision people [15].

In this study, we do not treat real color vision defect, but we create a virtual restricted color vision character who has a different color perception from normal color vision people. Moreover, we investigate an effect of role-play where participants must read the mind of a character with restricted color vision.

### Hypotheses

- The role-play group will respond with equal accuracy whether the communication partner has restricted color vision or not. The no-role-play group will make more errors when the communication partner has restricted color vision, than when the communication partner has normal color vision.
- The role-play group will respond more quickly than the no-role-play group, when the communication partner has restricted color vision.

## Method

### Ethical statement

The ethics committee at the Graduate School of Education, Kyoto University specifically approved this study. All participants provided written informed consent.

### Participants

Participants were recruited from Kyoto University, Japan. Forty university students with normal color vision (mean age: 21.5 years old, range 18–31 years old, 20 males and 20 females) were allocated into two groups: role-play group (mean age: 21.2 years old, range: 18–31 years old, 10 males and 10 females) and no-role-play group (mean age 21.9 years old, range: 18–30 years old, 10 males and 10 females). They took part in this experiment individually, in a psychological laboratory.

### Experimental design

A mixed model experimental design was used: role-play condition (between: role-play vs. no-role-play) × communication partner condition (within: restricted color vision vs. normal color vision).

### Director task

**Figure 1:**
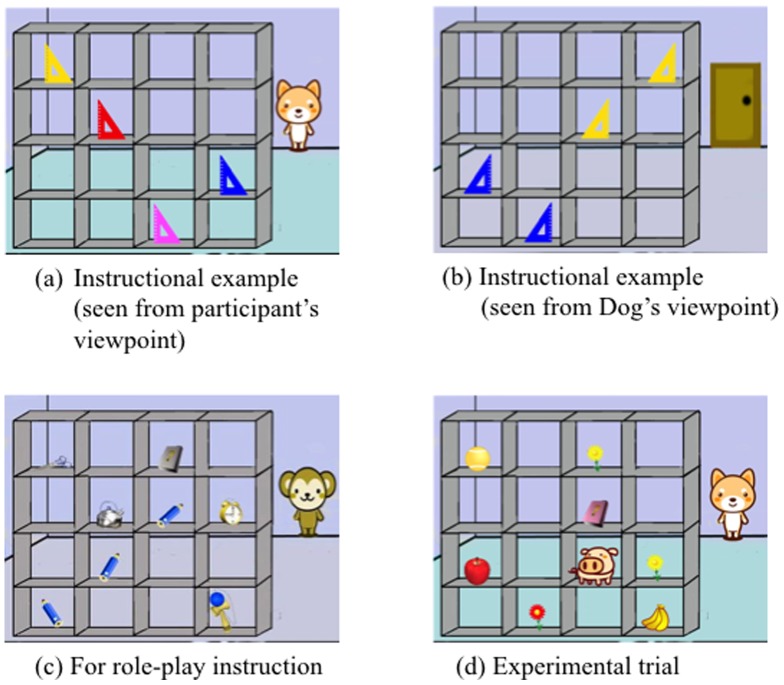
Examples of stimuli. (a–b) Images used to explain the restricted color vision condition to the participants: from participants view, they can see four different color of triangles (a) but a partner (Dog) see them as different colors (b). (c) Example of an role-play and no-role-play instruction. Participants saw restricted color vision during these instructions. (d) Example of a test trial. The participant heard the voice stimuli “Can I have the lower yellow flower?” if the participant cannot read the mind of the partner with restricted color vision, the participant would choose a yellow flower on the second slot from the bottom, but the partner see a red flower as a yellow flower. So, the participant should choose the red flower instead of the yellow flower.

We used two laptops for the Director Task. A normal laptop (SONY VAIO VPCEA1AFJ) was used to present the instructions to the participants, including role-play instruction and no-role-play instruction. Another laptop with a touch screen (FUJITSU LIFEBOOK AH/R3) was used for practice trials (10 trials) and test trials (40 trials). We made and edited picture stimuli using Adobe Photoshop, and recorded voice stimuli by IC recorder (Sony ICD-SX850). We used Microsoft PowerPoint 2007 to make the materials for the role-play instructions and no-role-play instructions and to present them. Test stimuli were made and presented by Super lab 4.0. A 4 type papers were used as instruction sheets for role-play instructions. Examples of picture stimuli are shown in Figure 1.

### First instruction for both groups

At first, the experimenter told the participants that they would be introduced to a “Department store game” and instructed in the rules of the game. The rules of “Department store game” were as follows; there were a manager and a clerk. The manager instructed the clerk to take an object and the clerk had to choose the object from a shelf with 4×4 slots. There were three players; participant, Monkey, and Dog. Then, Monkey had normal color vision like the participants, whereas Dog had restricted color vision in contrast to the participants. This information was explained using examples repeatedly. For example, Dog saw as yellow objects that the participants saw as red and he saw as blue objects that the participants saw as purple. Initially, participants had to answer the color question to confirm that they were people with normal color vision. After the instructions, the role-play group had an experience with role-play whereas the no-role-play group only heard some additional instructions from the experimenter.

### Role-play instruction for role-play group

Role-play participants played the role of the Dog manager with restricted color vision. They had to tell the Monkey clerk the instruction according to the instruction sheets, written as “Can I have…?” There were five instruction sheets, and they included either the size (big or small) or the place (upper or lower) and color (blue or yellow) and the name of the object (e.g. pencil, scissors). In the role-play instructions, restricted color vision pictures were presented, because the participants played the role of the Dog manager with restricted color vision. Monkey's responses were presented by an animation. He responded correctly only in response to the third instruction sheet, according to the previous research (c.f., [10]). After five instructions had been completed, the experimenter asked participants whether Monkey responded correctly. All the participants answered that Monkey took incorrect objects in response to almost all instructions.

### No-role-play instruction for no-role-play group

The experimenter presented the same picture stimuli and animations as the role-play instructions to participants, however, participants only heard instructions from an experimenter, without role-play experience. Participants see the Dog's restricted color world, and there is no difference about the visual experience between the role-play and no-role-play groups. The information included in this instruction matched the role-play instruction. The only difference between the groups in these instructions was whether the participants experienced the role and communicated with the Monkey.

### Second instruction for both groups

Then the experimenter told the participants that they would play the clerk role and Monkey and Dog would play the manager role next. The experimenter made sure the participants understood that Monkey had normal color vision whereas Dog had restricted color vision. The experimenter also emphasized that participants were required to respond according to the manager's instruction: Dog manager saw the colors of objects differently; thus, participants had to work out his intentions (i.e., mindread), according to his different perspective whereas all objects were in the same colors as those seen by Monkey manager.

### Practice trial for both groups

In the practice trial, presented picture stimuli include a patrol car and an airplane on a shelf with 4×4 slots, and at the same time, a woman's voice saying “patrol car” or “airplane” was presented. Participants had to touch the correct object according to the voice. Only when participants responded correctly, was the next trial presented. The practice trial consisted of 2 sets. Each set included 5 trials. These practice trials were for participants' practice of touch response, so when a first set had been done, the experimenter gave some feedback to participants about touch response. All participants were able to respond correctly when they finished the practice trials.

### Test trial for both groups

The test trial consisted of two conditions: 20 trials of normal color vision communication partner (Monkey) and another 20 trials of restricted color vision communication partner (Dog). We adopted a block design and did not mix two conditions within blocks. One block consisted of 10 trials of either normal color vision condition or restricted color vision condition. Two types of blocks were presented in turn, and which type was presented first was randomized between participants. When the block changed, instructions about the next trial were shown on the laptop (e.g. Dog is a manager in next block). In the pictures of test trials, there were eight objects on a shelf with 4×4 slots. The group of objects consisted of 2 blue and 1 purple object, and five unrelated objects (or 2 yellow and 1 purple object, and five unrelated objects) (see Figure 1). A woman's voice stimuli, which were different from the voice stimuli for the practice trial, was presented as “Monkey's voice” and a man's voice was presented as “Dog's voice.” We presented test stimuli by using a touch screen on the laptop computer. All the instructions from Monkey manager and Dog manager included either the size (big or small) or the place (upper or lower) and color (blue or yellow) and the name of object (e.g. pencil, scissors). All the contents of stimuli presented in normal color vision condition corresponded to those presented in restricted color vision condition.

In the restricted color vision condition, some trials did not require mindreading of a character with restricted color vision. For example, when Figure 1 (d) was presented and the voice stimulus said “Can I have the upper yellow flower?” an upper yellow flower for participants and for Dog manager with restricted color vision were identical, whereas when the same picture was presented and the voice stimulus said “Can I have the lower yellow flower?” there was a difference between them. We designed these trials that did not require mindreading, to check whether participants understood the task. All participants made less than two errors in trials not requiring mindreading. These trials were omitted when we analyzed the experimental data.

### Interview

After the test trials, the experimenter asked participants whether this game was difficult or not, whether participants came up with a strategy for the game, and what they were thinking when they played the game. Participants answered and the experimenter wrote down their answers. We designed this interview to check if participants used a strategy that did not require mindreading, however, none of the participants used a strategy that did not require mindreading.

## Results

### Error rates

**Figure 2:**
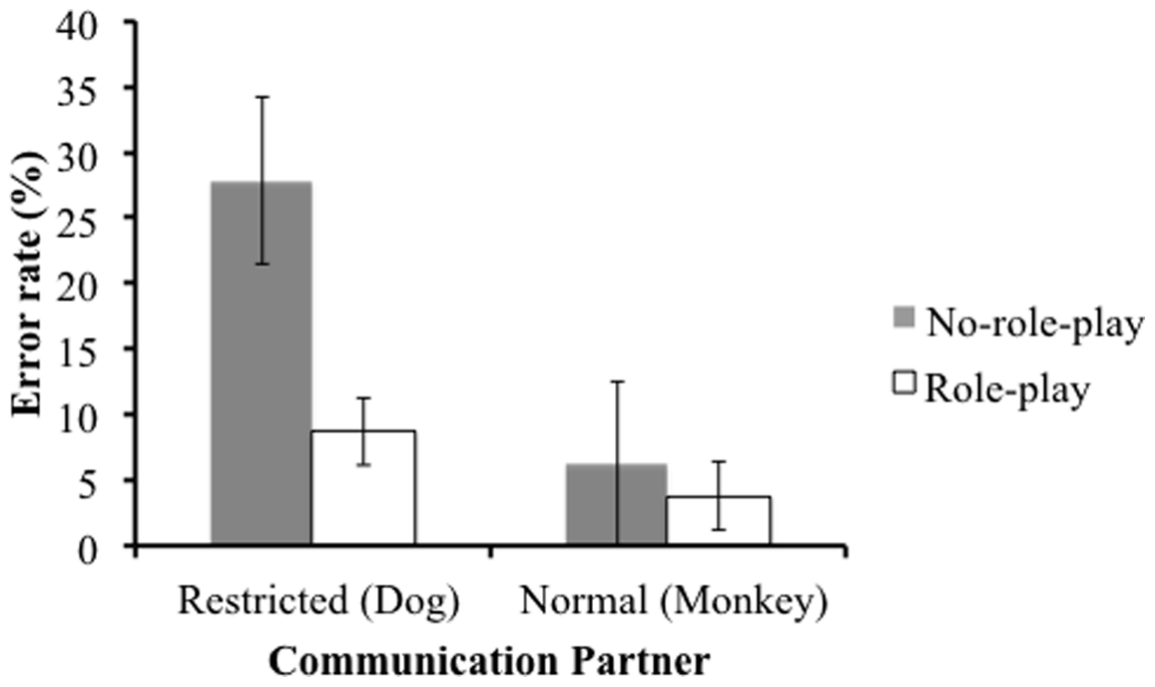
Mean (and 95% CI) of error rates.

Figure 2 shows means of error rates for all trials that required mindreading in the restricted color vision condition (16 trials) and corresponding trials in the normal color vision condition (16 trials). The experimental data were analyzed using a 2×2 mixed-design ANOVA using role-play condition (between: role-play vs. no-role-play) and communication partner condition (within: restricted color vision vs. normal color vision) to examine differences in the error rate. Both main effects and its interaction were significant (role-play: (*F* (1, 38) = 15.03, *p*<.001, 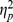 = .28, communication partner: *F* (1, 38) = 18.84, *p*<. 001, 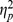 = .33, and the interaction: *F* (1, 38) = 7.33, *p* = .010, 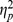 = .16). According to the *post-hoc* analyses, no-role-play participants made significantly more errors in the restricted color vision condition than in the normal color vision condition (*t* (19) = 3.85, *p* = .001 (two-tailed), *r* = .66), whereas among role-play participants, there was no difference between the conditions (*ns*).

### Reaction time

Only participants' correct responses were analyzed for reaction time data. In the practice trial, there were no significant differences between groups (*t* (38) = −.68, *ns*). That is, the ability to use the touch screen was not different between groups.

**Figure 3:**
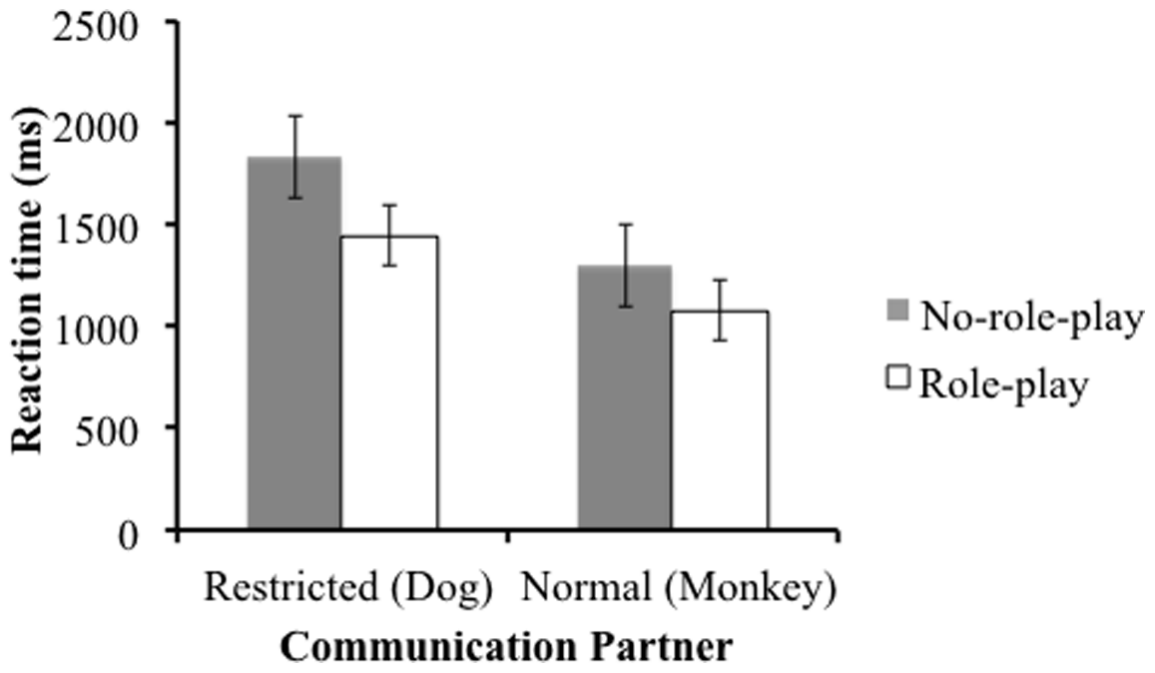
Mean (and 95% CI) of reaction times.

The same analyses were conducted for reaction time data. Figure 3 shows the means of reaction time. Both main effects were significant (role-play: (*F* (1, 38) = 4.41 *p* = .042, 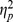 = .10, communication partner: *F* (1, 38) = 37.92, *p*<. 001, 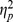 = .50). The role-play group responded significantly more quickly than the no-role-play group. In addition, participants responded significantly more quickly in the normal color vision condition compared to the restricted color vision condition. The interaction was not significant (*F* (1, 38) = 1.36, *ns*, 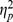 = .04).

## Discussion

In this study, we investigated the ability of adults to read the minds of people with restricted color vision. Our critical trials required participants to interpret another's instructions based on that person's color perception. All participants could understand that Monkey's color perception was the same as their own, whereas Dog's color perception was different to their own. During the test trials, however, the no-role-play group made more errors when the communication partner had restricted color vision than when the communication partner had normal color vision. On the other hand, the role-play group could respond accurately even if a communication partner had restricted color vision. This supports hypothesis A, “the role-play group will respond with equal accuracy whether the communication partner has restricted color vision or not. The no-role-play group will make more errors when the communication partner has restricted color vision, than when the communication partner has normal color vision.” This result is consistent with previous studies (e.g., [10]) in which the role-play group responded accurately when the communication partner could not see all of the objects, because some of them were physically occluded.

The practice trial reaction time result indicates that there is no difference of touch skill between the groups. Thus, test trial reaction time results show the effect of role-play on reading the mind of the communication partner. The role-play group responded more quickly than the no-role-play group in both conditions. This partly supports hypothesis B, “the role-play group will respond more quickly than the no-role-play group, when the communication partner has restricted color vision.” This result is inconsistent with previous research, which suggests that the role-play group will respond more quickly only when there is a difference of mental state between participants and communication partner [10]. The current experiment used a block design model, so participants had to switch mindreading approach depending on the communication partner, when blocks were changed. In the interview, some no-role-play participants said that after the Dog (with restricted color vision) trials, the Monkey (normal color vision) trials were difficult. On the other hand, none of the role-play group gave answers of this kind. It could be more difficult for the no-role-play group to switch mindreading approach. Many mindreading studies have addressed the relationship between mindreading and executive functions. Lin et al. [13] pointed out that the individual differences in working memory affects performance on the Director Task. For our novel Director Task, we also assume that executive function underlies the participants' performance. For the original Director Task, participants can use the occlusion as a cue to find out the director's different perspective when the stimuli are presented. On the other hand, the cues for this novel Director Task, (Dog has restricted color vision and Monkey has normal color vision), might be more difficult for participants to use. We consequently believe that the novel Director Task will be associated with a greater executive load. Moreover, when participants understand the perception of the person with restricted color vision, they have to inhibit their color perception and then take the restricted color perspective. Once again this implies increased executive load for this novel Director Task. Participant reports suggest that executive function is implicated in, role-play. Our study, however, did not investigate the relationship between the role-play effect and executive function or the individual differences of executive function in this color version Director Task. We plan to explore this further in a future study. Dumontheil, Apperly, and Blakemore [4] compared the trajectory of development of executive function and that of mindreading using a ‘director condition’ and a ‘no director condition’. In ‘director condition’, participants had to read the mind of the director standing at the opposite side of the shelf from where, some of the objects cannot be seen. In ‘no director condition’, the director was not present and participants were instructed not to touch the objects with a grey background. Dumontheil Apperly, and Blakemore [4] suggested that participants have to retain the rule that they should inhibit choice of objects with a grey background when the director is not present, so working memory skills are required for this condition. Dumontheil, Apperly, and Blakemore [4] found that the performance of adolescent participants was similar to that of adult participants in ‘no director condition’, on the other hand there was significant difference between the performance of adolescent participants and that of adult participants in ‘director condition’.

These results suggest that role-play activates mindreading among participants, in order to understand people with restricted color vision. When people infer another's mind, they can take another's perspective automatically when the situation is very simple [6], however, when the situation is complex, people are likely to respond based on a self-centered perspective and then inhibit it to take the perspective of another person[3], [4], [7], [13].

Recently, it has been suggested that spontaneous theory of mind is important for daily life mindreading [16]. We implicitly think, however, that others have the same perceptions and characteristics as ourselves. Thus, we read others' mind by using the strategy that all people will think as we do. Previous studies show that there are various characteristics in daily life (e.g., [17]–[18]). This present study suggests that role-play can decrease the discrepancies caused by differences in perception and characteristics.

Most mindreading tasks have created discrepancies by presenting situations like these:“A first placed an object into a box, then A left the scene, B moved the object to another box, then A returned” [19], and “A knows the contents of a box, but B does not know” [20]. Participants can see all of objects but Director cannot see some of them because of occlusion of the shelves (c.f., [3]–[5], [10], [13]). In this study, we investigate the ability to read the mind of people with different perception even when they see the same scene. Previous Director Tasks created mental discrepancies by controlling the situations (set occlusions to some slots). Compared to them, our novel Director Task makes mental discrepancies based on personal differences: A has normal color vision, and B has restricted color vision. This study has implications for research on mindreading and theory of mind. Furthermore, this study adds to what is known about how we understand people with different characteristics.

## References

[1] Wimmer H, Perner J (1983) Beliefs about beliefs: Representation and constraining function of wrong beliefs in young children's understanding deception. Cognition 13: 103–128. 6681741 10.1016/0010-0277(83)90004-5

[2] Perner J, Wimmer H (1985) “John thinks that Mary thinks that…”: Attribution of second-order beliefs by 5- to 10-year-old children. Journal of Experimental Child Psychology 39: 437–471.

[3] Keysar B, Barr DJ, Balin JA, Brauner JS (2000) Taking perspective in conversation: The role of mutual knowledge in comprehension. Psychological Science 11: 32–38. 11228840 10.1111/1467-9280.00211

[4] Dumontheil I, Apperly IA, Blakemore SJ (2010) Online usage of theory of mind continues to develop in late adolescence. Developmental Science 13: 331–338. 20136929 10.1111/j.1467-7687.2009.00888.x

[5] Dumontheil I, Küster O, Apperly IA, Blakemore SJ (2010) Taking perspective into account in a communicative task. NeuroImage 52: 1574–1583. 20510369 10.1016/j.neuroimage.2010.05.056

[6] Samson D, Apperly IA, Braithwaite JJ, Andrews BJ, Scott SEB (2010) Seeing it their way: Evidence for rapid and involuntary computation of what other people see. Journal of Experimental Psychology: Human Perception and Performance 36: 1255–1266. 20731512 10.1037/a0018729

[7] Surtees ADR, Butterfill SA, Apperly IA (2012) Direct and indirect measures of level-2 perspective-taking in children and adults. British Journal of Developmental Psychology 30: 75–86. 22429034 10.1111/j.2044-835X.2011.02063.x

[8] Flavell JH, Everett BA, Croft K, Flavell ER (1981) Young children's knowledge about visual-perception –further evidence for the Level 1-Level 2 distinction. Developmental Psychology 17: 99–103.

[9] Apperly IA (2011) Mindreaders. New York: Psychology Press.

[10] Furumi F, Koyasu M (2012) Does experience with role play activate "mindreading" in a perspective-taking task? The Japanese Journal of Psychology 83: 18–26 (in Japanese with English abstract). 22715535 10.4992/jjpsy.83.18

[11] Komeda H, Kawasaki M, Tsunemi K, Kusumi T (2009) Differences between estimating protagonists' emotions and evaluating readers' emotions in narrative comprehension. Cognition & Emotion 23: 135–151.

[12] Komeda H, Kosaka H, Saito DN, Inohara K, Munesue T, et al.. (2013) Episodic memory retrieval for story characters in high-functioning autism. Molecular Autism 4. doi:10.1186/2040-2392-4-20. 10.1186/2040-2392-4-20 PMC3695882 23800273

[13] Lin S, Keysar B, Epley N (2010) Reflexively mindblind: Using theory of mind to interpret behavior requires effortful attention. Journal of Experimental Social Psychology 46: 551–556.

[14] Birch J (2012) Worldwide prevalence of red-green color deficiency. Journal of the Optical Society of America A 29: 313–320. 10.1364/JOSAA.29.000313 22472762

[15] Shepard RN, Cooper LA (1992) Represetation of colors in the blind, color-blind, and normally sighted. Psychological Science 3: 97–104.

[16] Senju A (2012) Spontaneous theory of mind and its absence in autism spectrum disorders. The Neuroscientist 18: 108–113. 21609942 10.1177/1073858410397208 PMC3796729

[17] Happé F (1999) Autism: Cognitive deficit or cognitive style? Trends in Cognitive Science 3: 216–222. 10.1016/s1364-6613(99)01318-2 10354574

[18] Oyserman D, Lee SWS (2008) Does culture influence what and how we think? Effects of priming individualism and collectivism. Psychological Bulletin 134: 311–342. 18298274 10.1037/0033-2909.134.2.311

[19] Baron-Cohen S, Leslie AM, Frith U (1985) Does the autistic child have a “theory of mind”? Cognition 21: 37–46. 2934210 10.1016/0010-0277(85)90022-8

[20] Perner J, Frith U, Leslie AM, Leekam SR (1989) Exploration of the autistic child's theory of mind: Knowledge, belief, and communication. Child Development 60: 689–700. 2737018

